# Supplementary material for: Tandemly duplicated CYP82Ds catalyze 14-hydroxylation in triptolide biosynthesis and precursor production in Saccharomyces cerevisiae
Source: Nat Commun. 2023 Feb 16;14:875. doi: 10.1038/s41467-023-36353-y (PMC9936527; doi:10.1038/s41467-023-36353-y)
Supplement: Supplementary file 1 — Supplementary Information [file 41467_2023_36353_MOESM1_ESM.pdf]

Tandemly duplicated CYP82Ds catalyze 14-hydroxylation in triptolide biosynthesis and precursor production in *Saccharomyces cerevisiae*

Zhang *et al.*

Supplementary Table 1. Information on the CYP82D family of genes in *Tripterygium wilfordii*.

Gene ID	Chromosome number	Coding amino acid number
TW06G01463.1	06	518
TW06G01464.1	06	518
TW08G01036.1	08	530
TW11G01090.1	11	529
TW11G01092.1	11	521
TW11G01093.1	11	530
TW12G01155.1 (CYP82D274)	12	533
TW12G01160.1 (CYP82D263)	12	528
TW12G01162.1	12	522
TW15G01388.1	15	528
TW19G00937.1	19	516

Supplementary Table 2. Different combinations of CYP728B70 and TwPOR.

MCS1	MCS2					
CYP728B70	POR1	tPOR1	POR3	tPOR3	POR4	tPOR4
tCYP728B70	POR1	tPOR1	POR3	tPOR3	POR4	tPOR4

Supplementary Table 3. *Saccharomyces cerevisiae* strains used in this study.

Strains	Genotype or characteristic	Resource
BY4741	*MATa; his3Δ1; leu2Δ0; met15Δ0; ura3Δ0*	ATCC
BY-HZ16	BY-T20/*rox1△erg9*::*△-218--175pyjl064w△ypl062w△*	[1]
BY4741H	BY4741/*rox1△erg9*::*△-218--175pyjl064w△ypl062w△*	Made by Tianyuan Hu
BY-PS2	BY-HZ16/*YPRC△15*::(P_{TDH3}-*SmKSL-SmCPS1*-T_{TPI1}-P_{ADH1}-*SmKSL-SmCPS1*-T_{PGI})	[2]
BY-ZY1	BY-HZ16/pYES2::(P_{TDH3}-*tSmKSL1-CfTPS1*-T_{TPI1}-P_{ADH1}-*CYP82D274*-T_{PGI})	This study
BY-ZY1H	BY-ZY1/pESC-LEU::(*CYP720B4*+*TwPOR3*)	This study
BY-ZY2D	Fused BY-HZ16 with BY4741H	This study
BY-ZY3	BY-HZ16/*YPRC△15*::(P_{TDH3}-*tSmKSL1-CfTPS1*-T_{TPI1}-P_{ADH1}-*CYP82D274*-T_{PGI}-P_{PGK1}-*TRP1*-T_{ADH1}-P_{TEF2}-*TwPOR3*-T_{CYC1})	This study
BY-ZY4	BY-HZ16/*YPRC△15*::(P_{TDH3}-*tSmKSL1-CfTPS1*-T_{TPI1}-P_{ADH1}-$CYP82D274^{L234M}$-T_{PGI}-P_{PGK1}-*TRP1*-T_{ADH1}-P_{TEF2}-*TwPOR3*-T_{CYC1})	This study
BY-ZY5	BY-HZ16/*YPRC△15*::(P_{TDH3}-*tSmKSL1-CfTPS1*-T_{TPI1}-P_{ADH1}-$CYP82D274^{G398S}$-T_{PGI}-P_{PGK1}-*TRP1*-T_{ADH1}-P_{TEF2}-*TwPOR3*-T_{CYC1})	This study
BY-ZY6	BY-HZ16/*YPRC△15*::(P_{TDH3}-*tSmKSL1-CfTPS1*-T_{TPI1}-P_{ADH1}-$CYP82D274^{M117A}$-T_{PGI}-P_{PGK1}-*TRP1*-T_{ADH1}-P_{TEF2}-*TwPOR3*-T_{CYC1})	This study
BY-ZY7	BY-ZY2D/*YPRC△15*::(P_{TDH3}-*tSmKSL1-CfTPS1*-T_{TPI1}-P_{ADH1}-*CYP82D274*-T_{PGI}-P_{TEF2}-*TwPOR3*-T_{CYC1})	This study
BY-ZY8	BY-ZY2D/*YPRC△15*::(P_{TDH3}-*tSmKSL1-CfTPS1*-T_{TPI1}-P_{ADH1}-$CYP82D274^{L234M}$-T_{PGI}-P_{TEF2}-*TwPOR3*-T_{CYC1})	This study
BY-ZY9	BY-ZY2D/*YPRC△15*::(P_{TDH3}-*tSmKSL1-CfTPS1*-T_{TPI1}-P_{ADH1}-$CYP82D274^{G398S}$-T_{PGI}-P_{TEF2}-*TwPOR3*-T_{CYC1})	This study
BY-ZY10	BY-ZY2D/*YPR+-C△15*::(P_{TDH3}-*tSmKSL1-CfTPS1*-T_{TPI1}-P_{ADH1}-$CYP82D274^{M117A}$-T_{PGI}-P_{TEF2}-*TwPOR3*-T_{CYC1})	This study
BY-ZY11H	BY-ZY3/pESC-LEU::(*CYP720B4*+*TwPOR3*)	This study
BY-ZY12H	BY-ZY4/pESC-LEU::(*CYP720B4*+*TwPOR3*)	This study
BY-ZY13H	BY-ZY5/pESC-LEU::(*CYP720B4*+*TwPOR3*)	This study
BY-ZY14H	BY-ZY6/pESC-LEU::(*CYP720B4*+*TwPOR3*)	This study
BY-ZY15D	BY-ZY7/pESC-LEU::(*CYP720B4*+*TwPOR3*)	This study
BY-ZY16D	BY-ZY8/pESC-LEU::(*CYP720B4*+*TwPOR3*)	This study
BY-ZY17D	BY-ZY9/pESC-LEU::(*CYP720B4*+*TwPOR3*)	This study
BY-ZY18D	BY-ZY10/pESC-LEU::(*CYP720B4*+*TwPOR3*)	This study

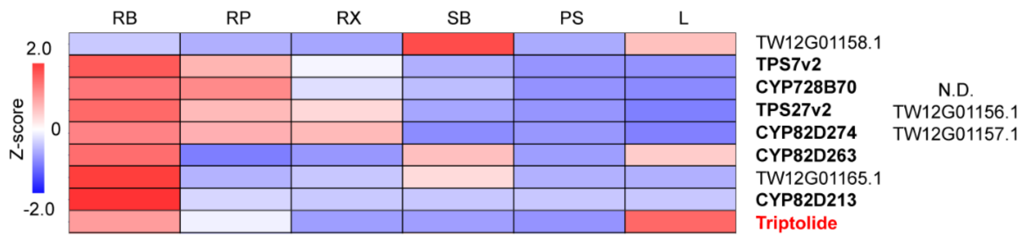

Supplementary Fig. 1. Coexpression profiles of CYP82Ds in Chr12 with previously characterized genes and triptolide. Root periderm (RB), root phloem (RP), root xylem (RX), stem vascular bundle (PS), stem periderm (SB), and leaf (L). N.D. indicates not detected.

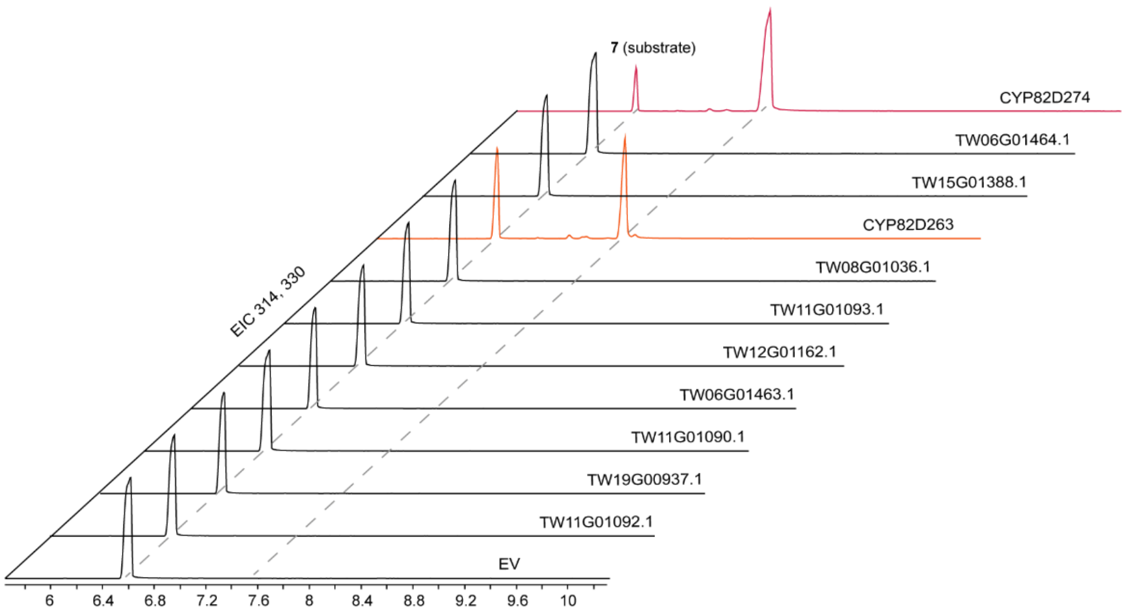

Supplementary Fig. 2. GC–MS of methylated products of CYP82Ds-catalyzed dehydroabietic acid (7).

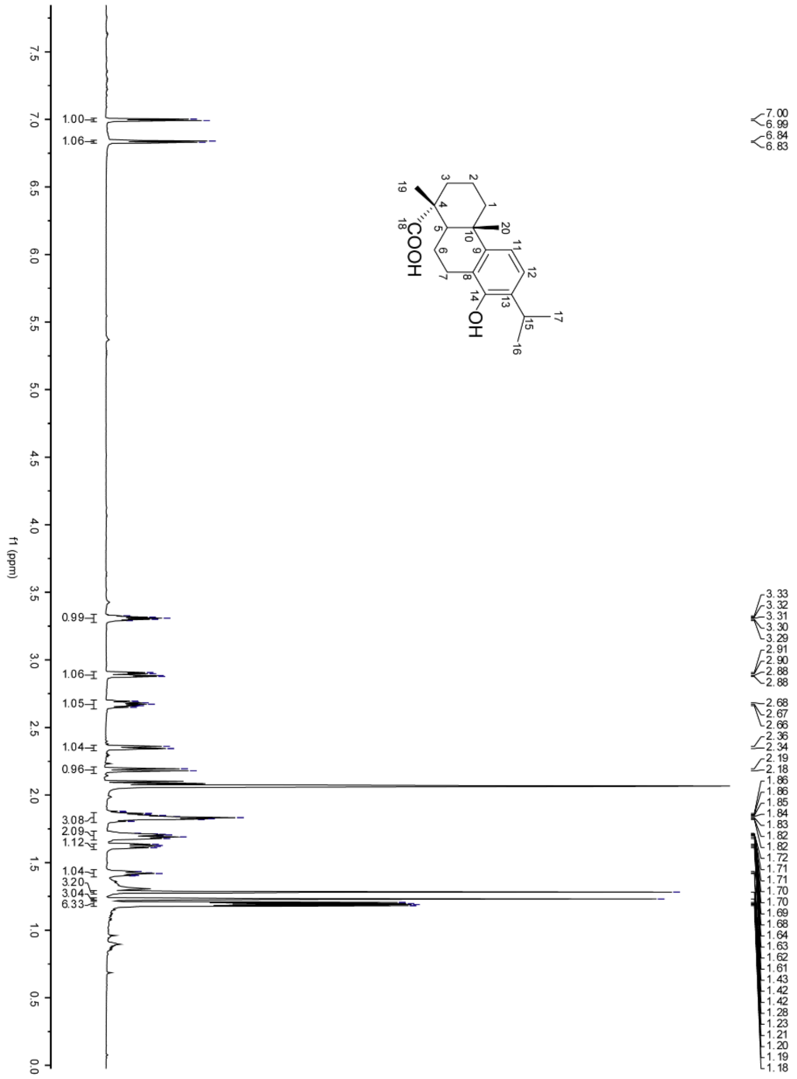

Supplementary Fig. 3. ^{1}H NMR spectrum of 14-hydroxy-dehydroabietic acid (11).

(C_3D_6O, 800 MHz).

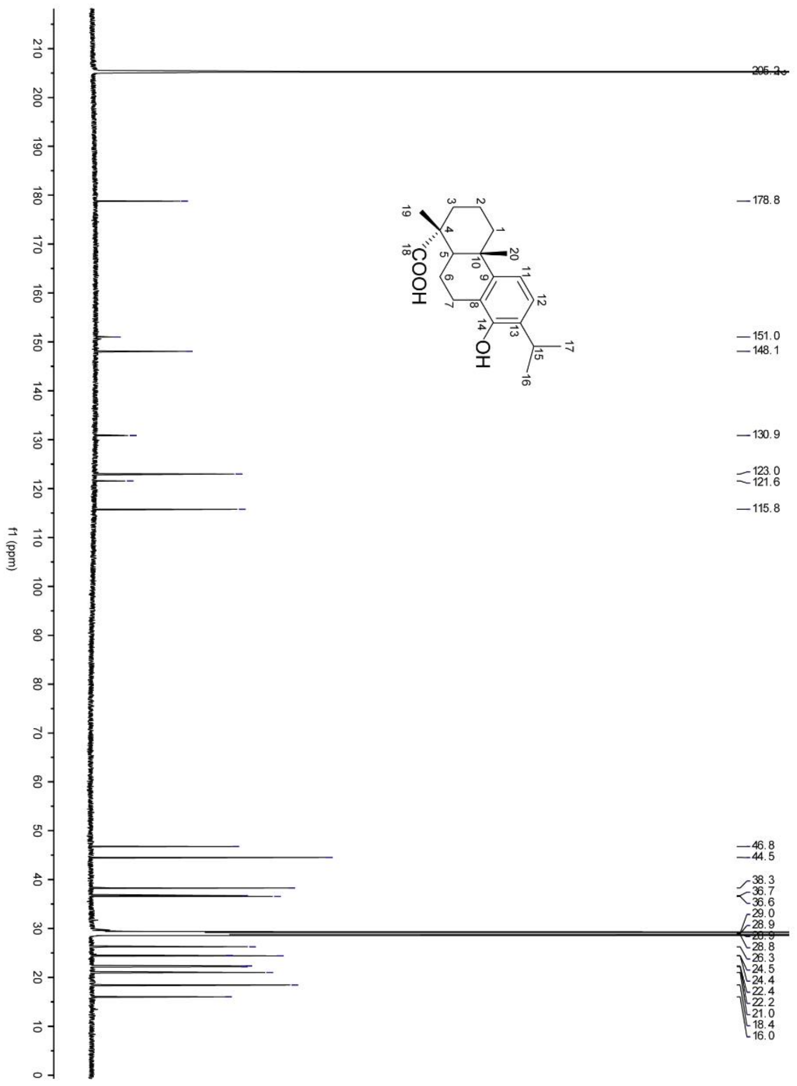

Supplementary Fig. 4. ^{13}C NMR spectrum of (11). (C_3D_6O, 200 MHz).

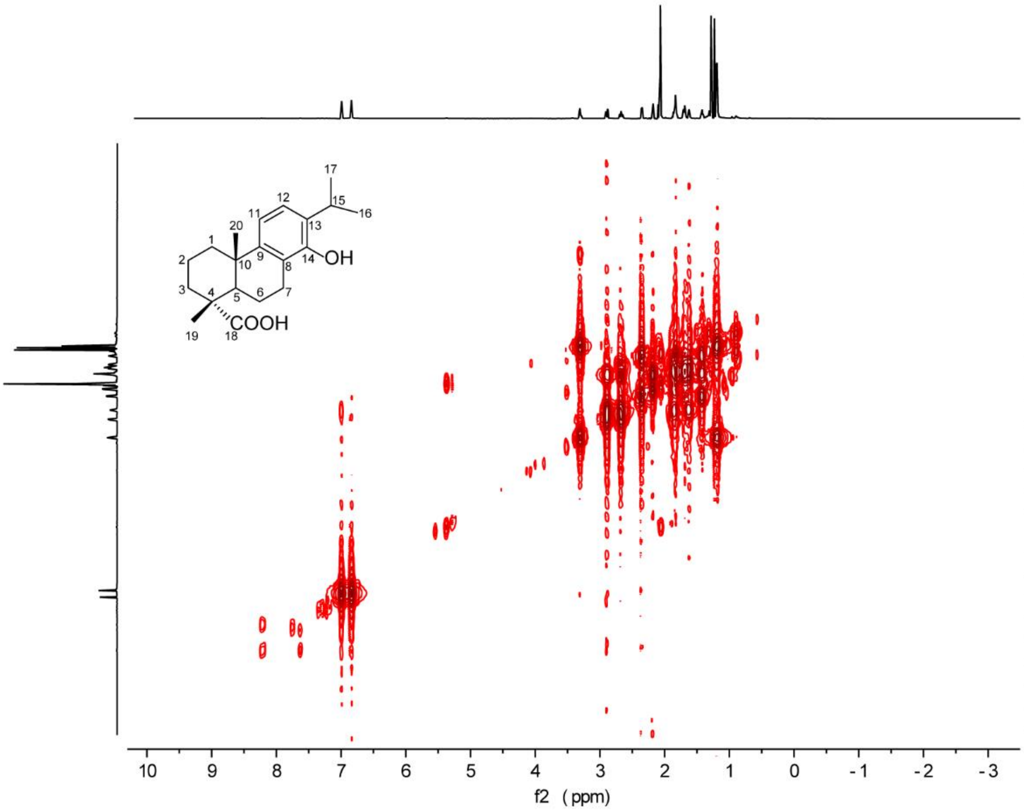

Supplementary Fig. 5. **^1H-^1H COSY spectrum of (11) in C_3D_6O.**

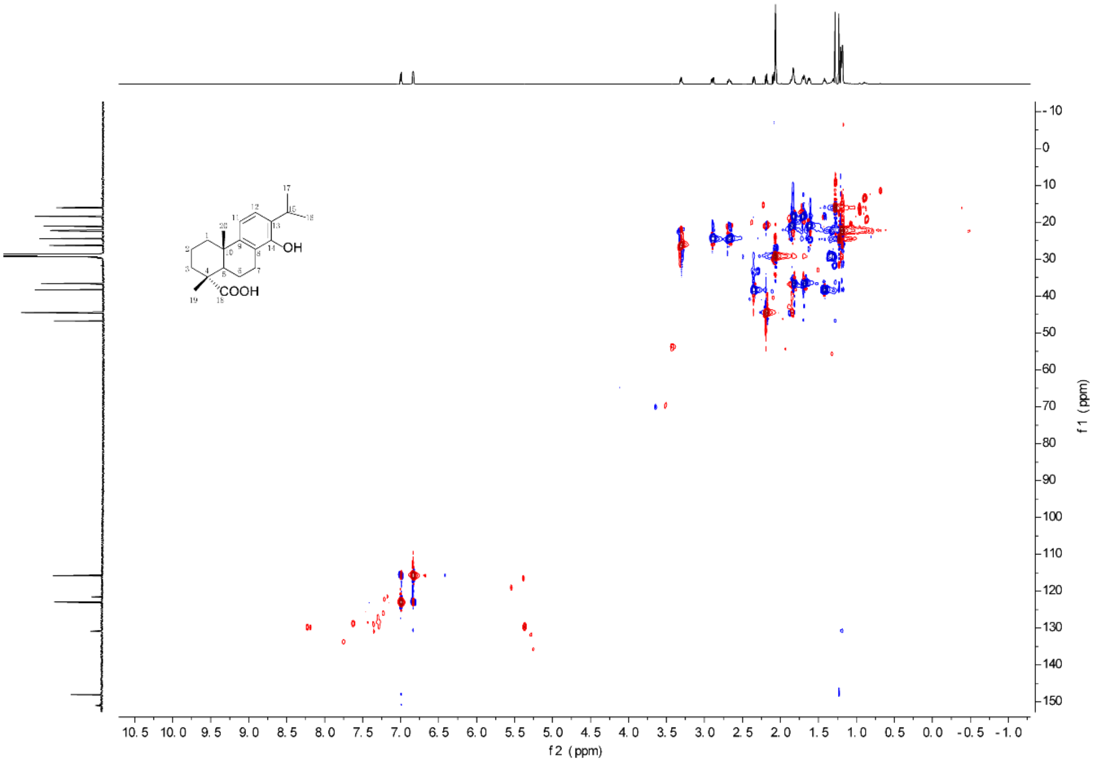

Supplementary Fig. 6. HSQC spectrum of (11) in C_3D_6O.

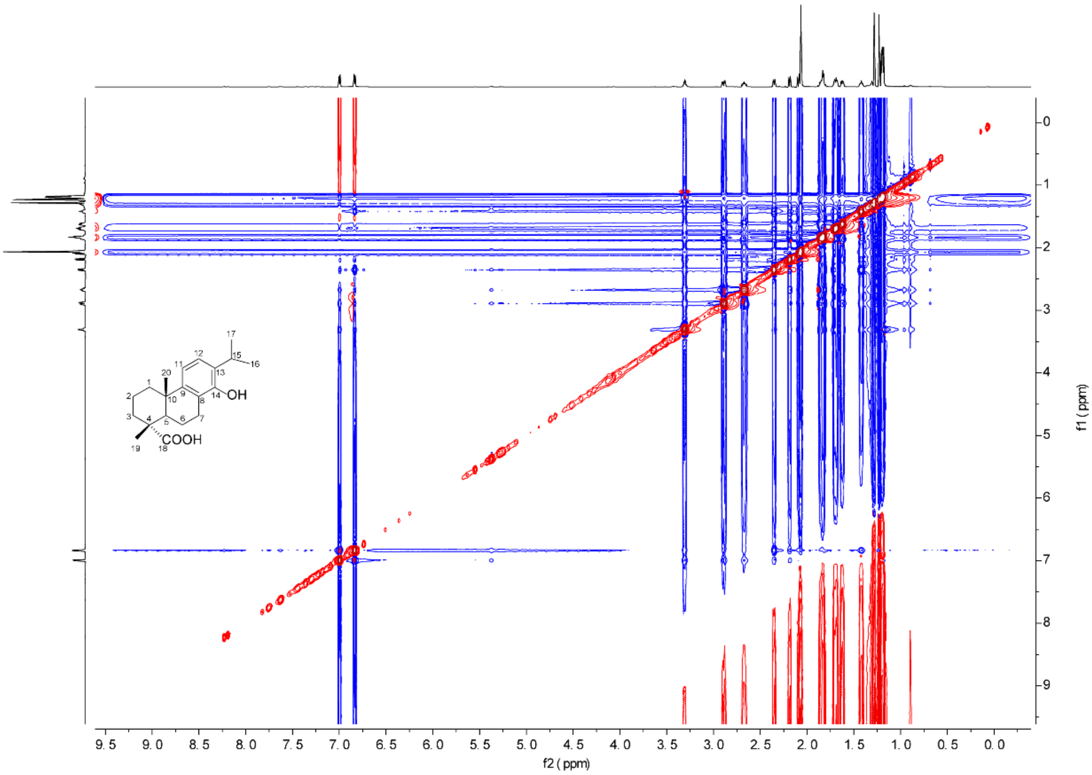

Supplementary Fig. 7. NOESY spectrum of (11) in C_3D_6O.

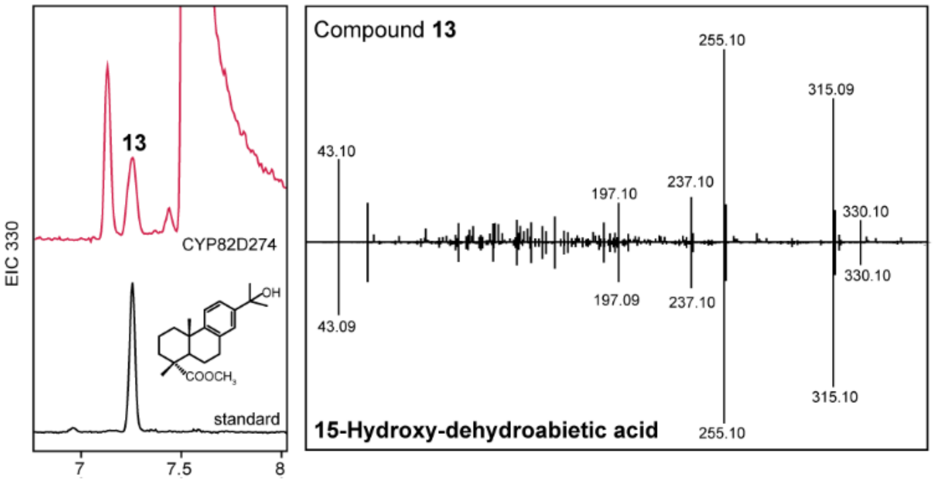

Supplementary Fig. 8. GC–MS analysis of compound (13) and the authentic standard 15-hydroxy-dehydroabietic acid. The purity of the authentic standard of **13** was > 98%.

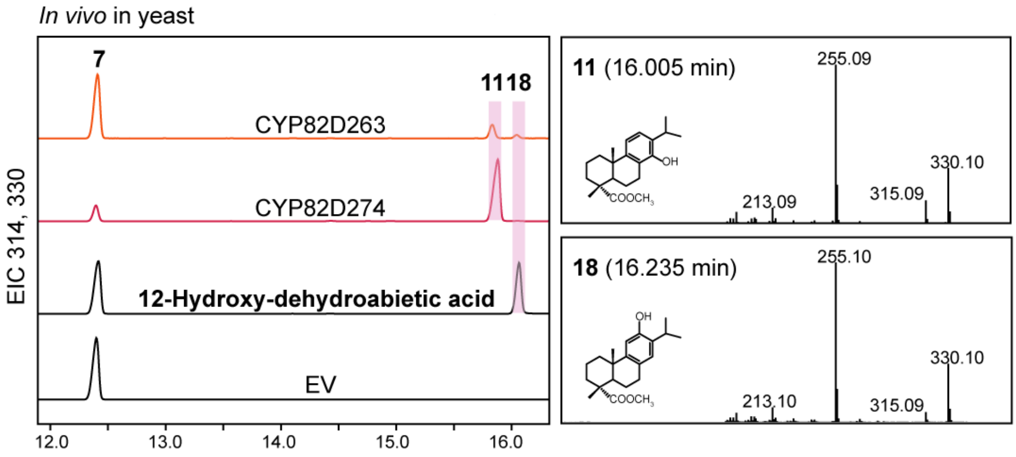

Supplementary Fig. 9. GC–MS analysis of methylated products of the reaction of (7) catalyzed by CYP82D274 (pink line) and CYP82D263 (orange line). Empty vector (EV) denotes yeast transformed with an empty vector without CYP. The GC–MS detection method was improved over that used to obtain the results in Fig. 3a, and the new product was identified as 12-hydroxy-dehydroabietic acid (**18**) [3].

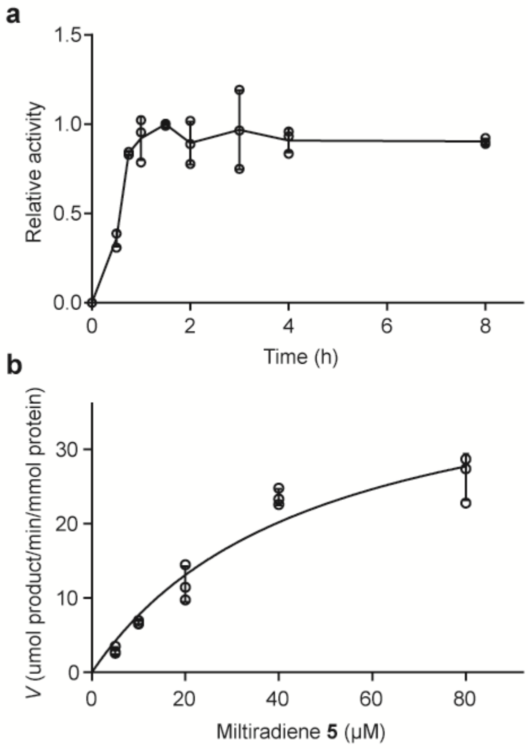

Supplementary Fig. 10. ***In vitro* assays. a** Effect of time on the activities of CYP82D274 for the reaction of **7**. **b** Kinetic profiles of CYP82D274 for the reaction of miltiradiene (**5**). Kinetic parameters were calculated with nonlinear regression analysis using the Michaelis–Menten model. Data are presented as mean values ± standard deviation from three biological independent replicates, and the black circles represent the individual data points. Source data are provided as a Source Data file.

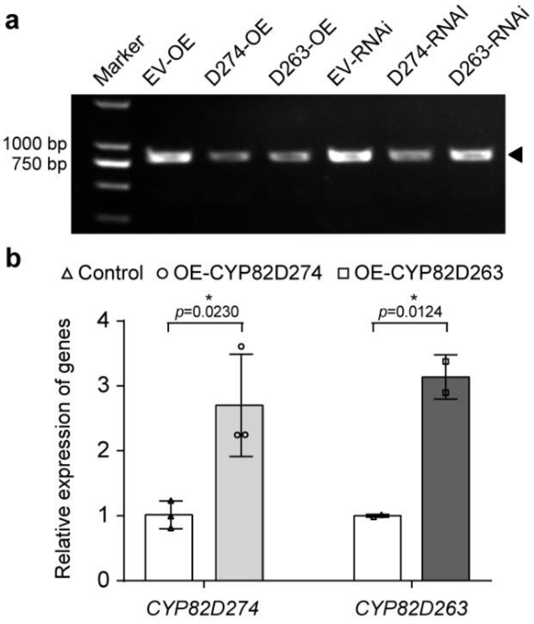

Supplementary Fig. 11. *In vivo* assays of *CYP82D274* and *CYP82D263* in plant cells. **a** Verification of successful gene transformation of cells. The black triangle indicates 798 bp electrophoretic bands amplified from expression vectors. Each group had three biological replicates with similar results. Partial electrophoresis results are shown above. **b** Relative expression of *CYP82D274* and *CYP82D263* in overexpression cell lines, analyzed by the $2^{-\triangle\triangle Ct}$ method. *EFLα* was designated as the housekeeping gene, and the empty vector group was assigned as the reference sample. Data are presented as mean values ± SD from three biological independent replicates (n=3, each biological replicate is composed of three technical replicates). A single asterisk indicates $P<0.05$ (two-sided Student's *t* test). Source data are provided as a Source Data file.

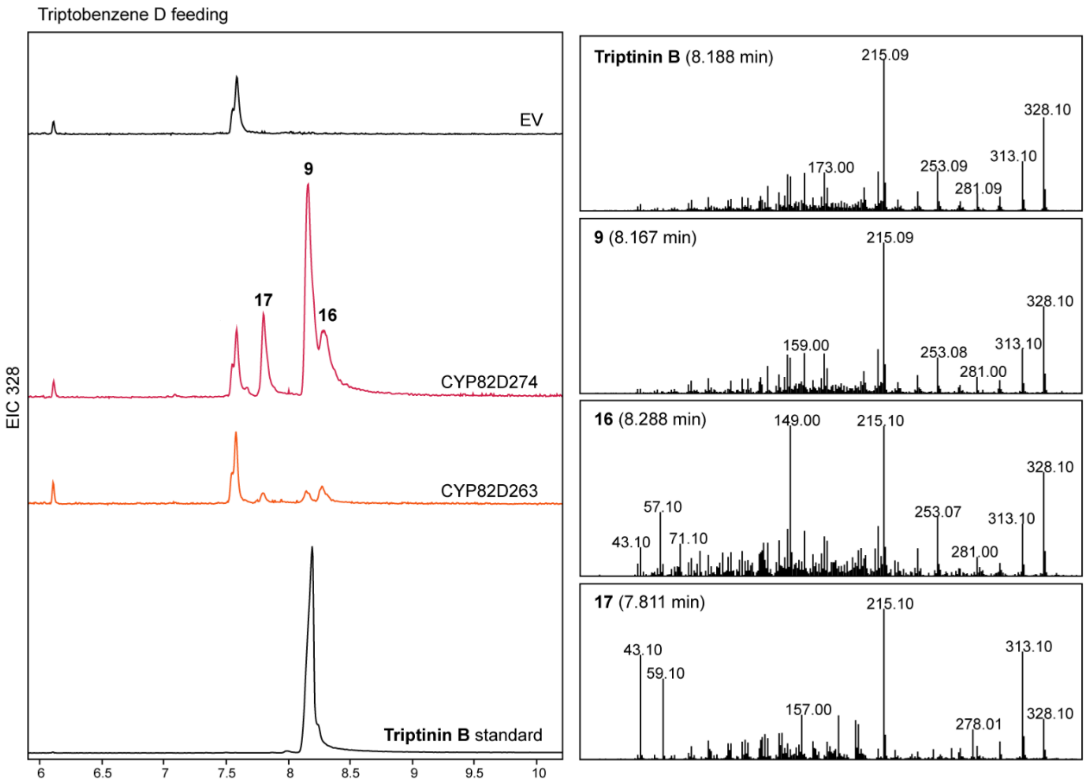

Supplementary Fig. 12. GC–MS analysis of methylated products *in vivo* with triptobenzene D (8) as the substrate. The purity of the authentic standard of triptinin B (**9**) was > 97%.

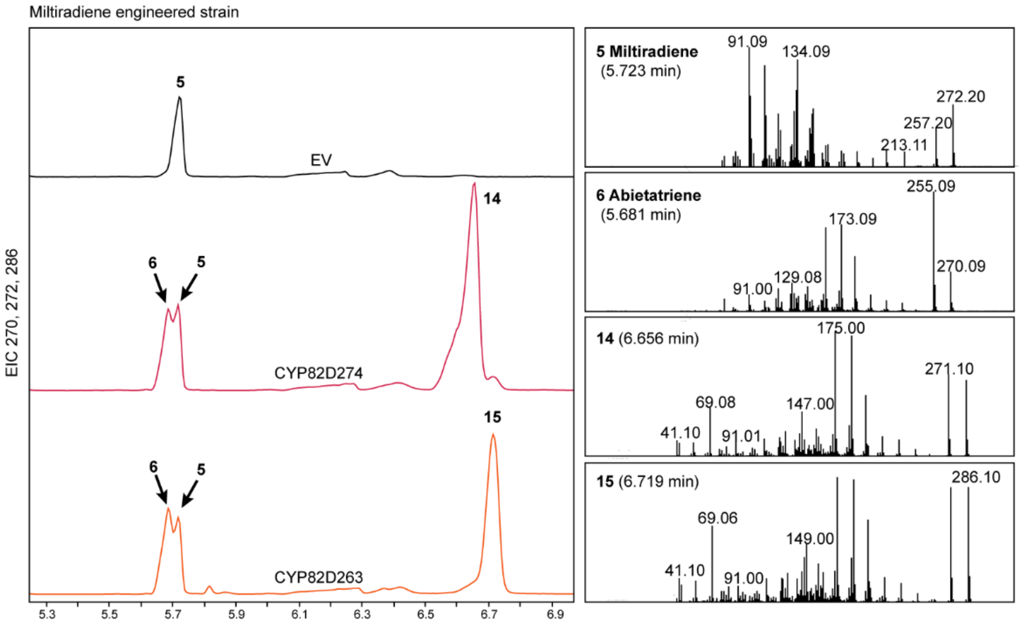

Supplementary Fig. 13. GC–MS analysis of products in miltiradiene (5) self-produced engineered yeast. Empty vector (EV) denotes yeast transformed with an empty vector without CYP.

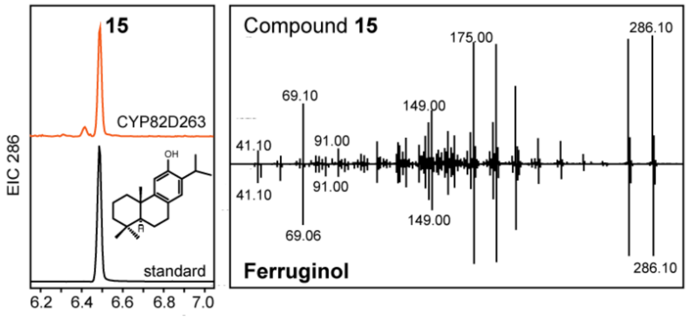

Supplementary Fig. 14. GC–MS analysis of (15) and the authentic standard ferruginol. The purity of the authentic standard of **15** was > 98%.

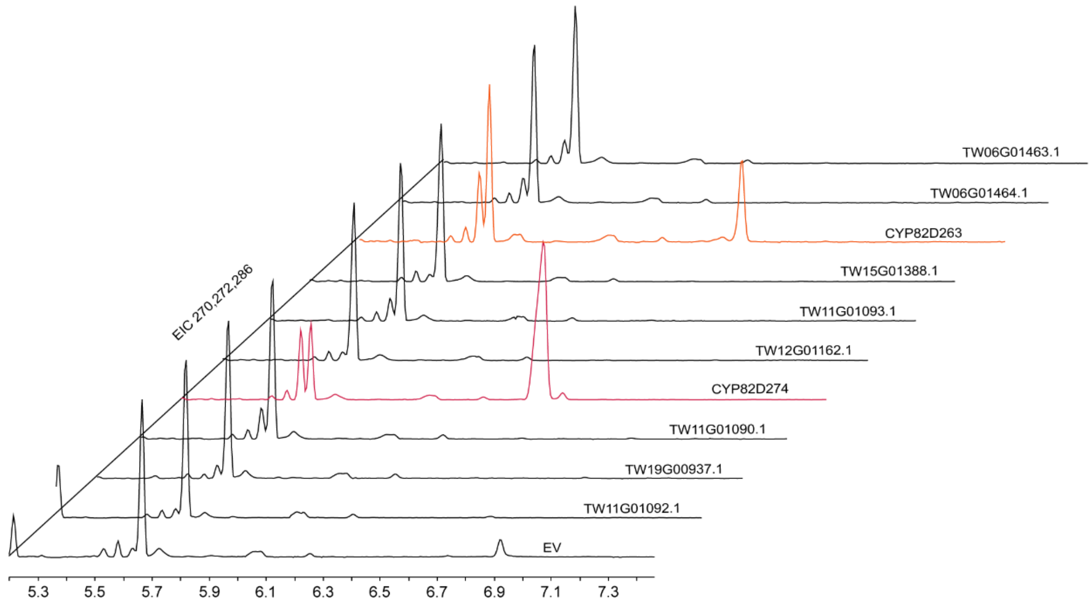

Supplementary Fig. 15. GC–MS analysis of the products of CYP82Ds-catalyzed miltiradiene (5).

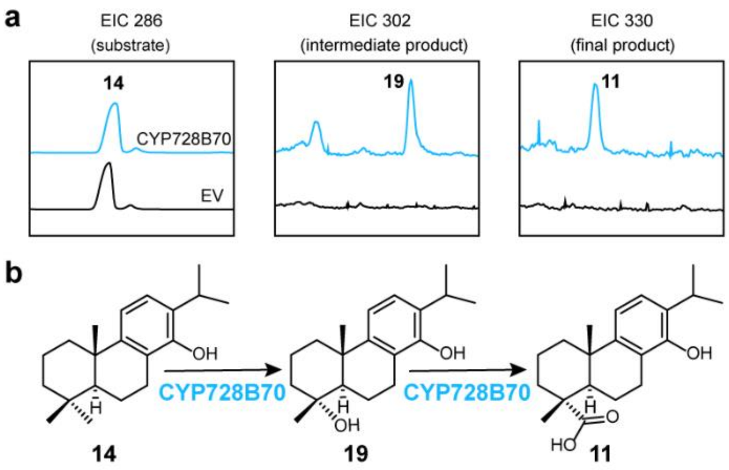

Supplementary Fig. 16. GC–MS analysis of CYP728B70 products *in vivo* with 14-hydroxy-abietatriene (14) as the substrate. a Extracted ion chromatogram of the CYP728B70 fermentation products. **b** Catalytic reaction processes.

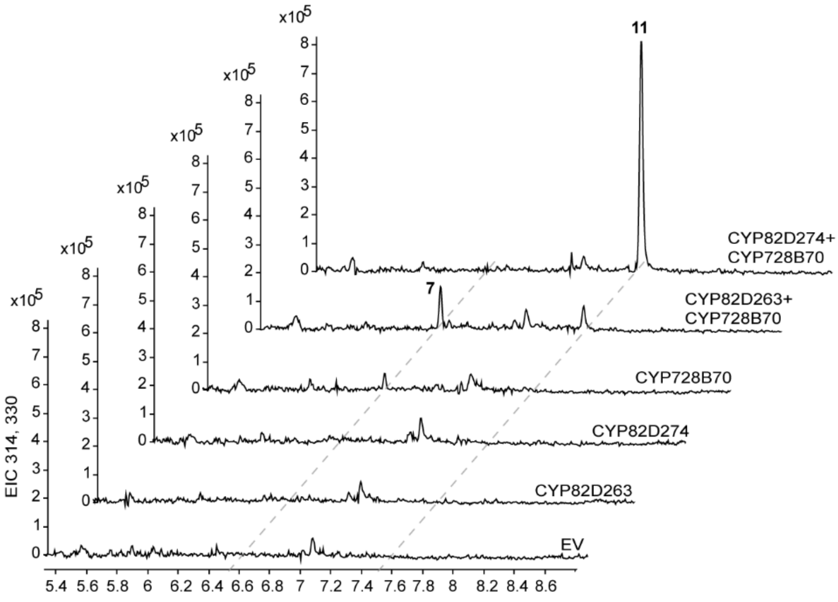

Supplementary Fig. 17. GC–MS analysis of methylated products generated by CYP82D274 and CYP82D263 coexpressed with CYP728B70.

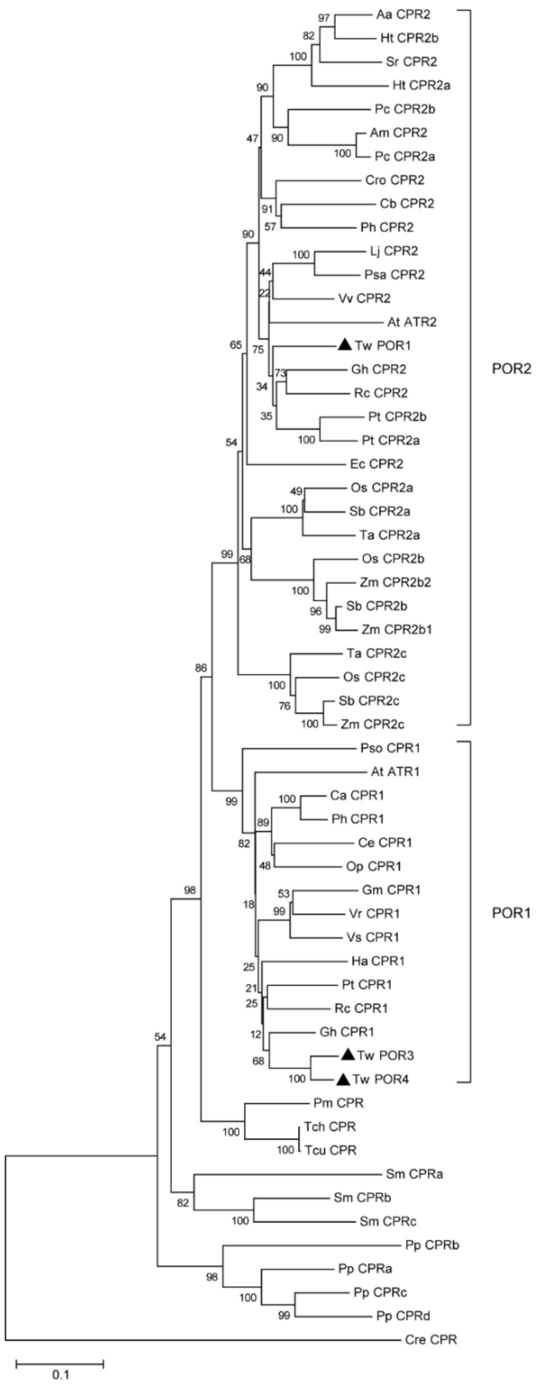

Supplementary Fig. 18. Phylogenetic analysis of 57 full-length plant POR sequences. The bootstrapped neighbor-joining tree was built in MEGA 6.0 software with maximum likelihood method (1000 bootstraps). The amino acid sequences can be found at http://www.p450.kvl.dk/. The PORs of *T. wilfordii* are marked with triangles.

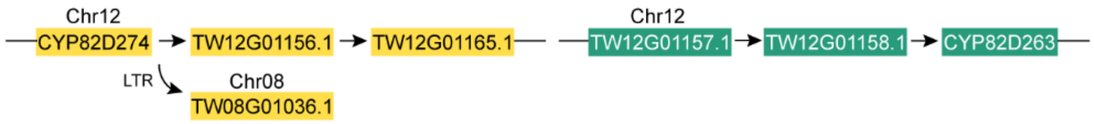

Supplementary Fig. 19. Diagram of the proposed gene replication events of CYP82Ds on Chr12. LTR indicates long terminal repeat element of retrotransposon. The arrows represent the replication order.

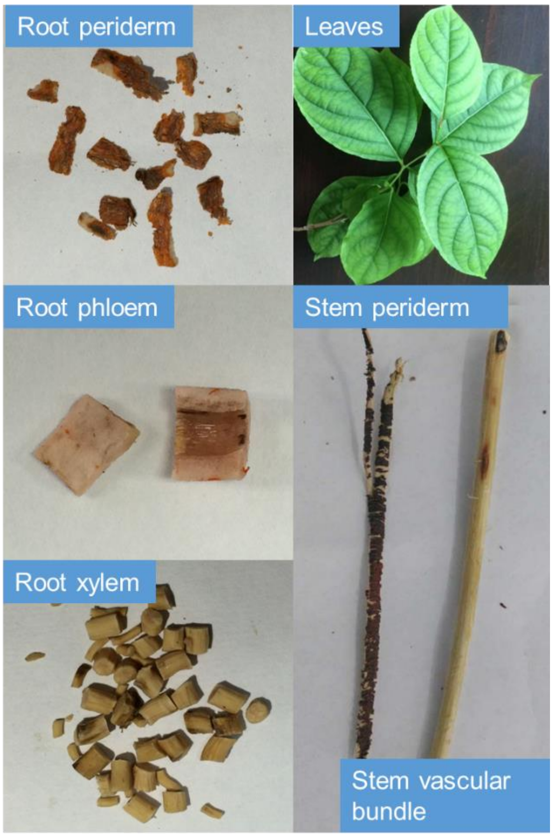

Supplementary Fig. 20. Six plant tissues of *T. wilfordii*. Root periderm (RB), root phloem (RP), root xylem (RX), stem vascular bundle (PS), stem periderm (SB), and leaves (L) were used for RNA-seq.

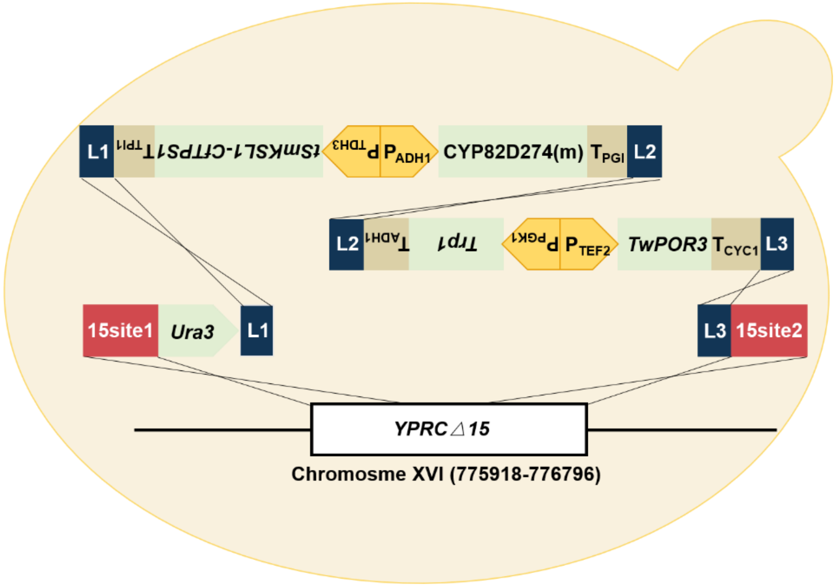

Supplementary Fig. 21. Schematic representation of the integration of target genes into the yeast genome by the modularized two-step chromosome integration technique [4].

Supplementary Note 1. NMR analysis.

14-Hydroxy-dehydroabietic acid (**11**) (3.0 mg, white amorphous powder)

^{1}H NMR (C_3D_6O, 800 MHz) δ 2.35 (1H, d, J = 12.6 Hz, H-1a), 1.44-1.40 (1H, m, H-1b), 1.72-1.67 (2H, m, H-2), 2.19 (1H, d, J = 12.6 Hz, H-4), 1.84 (3H, m, H-6a), 1.62 (1H, dd, J = 13.2, 8.0 Hz, H-6b), 3.31 (1H, m, H-7), 6.84 (1H, d, J = 8.0 Hz, H-11), 7.00 (1H, d, J = 8.0 Hz, H-12), 1.19 (6H, dd, J = 13.2, 6.9 Hz, H-15, 19), 1.23 (3H, s, H-16), 2.71-2.63 (1H, m, H-17a), 2.89 (1H, dd, J = 17.2, 6.8 Hz, H-17b), 1.28 (3H, s, H-20); ^{13}C NMR (C_3D_6O, 200 MHz) δ 38.3 (C-1), 18.4 (C-2), 36.6 (C-3), 44.5 (C-4), 46.8 (C-5), 21.0 (C-6), 26.3 (C-7), 121.6 (C-8), 148.1 (C-9), 36.7 (C-10), 115.8 (C-11), 123.0 (C-12), 130.9 (C-13), 151.0 (C-14), 22.2 (C-15), 24.4 (C-16), 24.5 (C-17), 178.8 (C-18), 22.4 (C-19), 16.0 (C-20) [5].

Supplementary references

1. Hu, T. *et al.* Engineering chimeric diterpene synthases and isoprenoid biosynthetic pathways enables high-level production of miltiradiene in yeast. *Metab. Eng.* **60**, 87-96 (2020).
2. Tu, L. *et al.* Genome of *Tripterygium wilfordii* and identification of cytochrome P450 involved in triptolide biosynthesis. *Nat. Commun.* **11**, 971 (2020).
3. Kinouchi, Y. *et al.* Potential antitumor-promoting diterpenoids from the stem bark of *Picea glehni*. *J. Nat. Prod.* **63**, 817-820 (2000).
4. Li, S., Ding, W., Zhang, X., Jiang, H. & Bi, C. Development of a modularized two-step (M2S) chromosome integration technique for integration of multiple transcription units in *Saccharomyces cerevisiae*. *Biotechnol. Biofuels* **9**, 232 (2016).
5. Zapata, B. *et al.* Cytotoxic, immunomodulatory, antimycotic, and antiviral activities of semisynthetic 14-hydroxyabietane derivatives and triptoquinone C-4 epimers. *MedChemComm* **4**, 1239 (2013).
