# Supplementary material for: Tandemly duplicated CYP82Ds catalyze 14-hydroxylation in triptolide biosynthesis and precursor production in Saccharomyces cerevisiae
Source: Nat Commun. 2023 Feb 16;14:875. doi: 10.1038/s41467-023-36353-y (PMC9936527; doi:10.1038/s41467-023-36353-y)
Supplement: Supplementary file 3 — Description of Additional Supplementary Files [file 41467_2023_36353_MOESM3_ESM.pdf]

Description of Additional Supplementary Files

File Name: Supplementary Data 1

Description: Synonymous substitution rates values and duplication/divergence times of genes.

File Name: Supplementary Data 2

Description: Relative content of diterpenoids in the control and CYP82Ds RNAi cell lines.

File Name: Supplementary Data 3

Description: Relative content of diterpenoids in the control and CYP82Ds-overexpressing cell lines.

File Name: Supplementary Data 4

Description: Relative content of diterpenoids in the control and substrate fed cell lines.

File Name: Supplementary Data 5

Description: All primers used in this study.

File Name: Supplementary Data 6

Description: Information of CYP82Ds.
