# Supplementary material for: Tandemly duplicated CYP82Ds catalyze 14-hydroxylation in triptolide biosynthesis and precursor production in Saccharomyces cerevisiae
Source: Nat Commun. 2023 Feb 16;14:875. doi: 10.1038/s41467-023-36353-y (PMC9936527; doi:10.1038/s41467-023-36353-y)
Supplement: Supplementary file 5 — Reporting Summary [file 41467_2023_36353_MOESM5_ESM.pdf]

nature portfolio

Corresponding author(s): Luqi Huang & Wei Gao

Last updated by author(s): Jan 26, 2023

Reporting Summary

Nature Portfolio wishes to improve the reproducibility of the work that we publish. This form provides structure for consistency and transparency in reporting. For further information on Nature Portfolio policies, see our Editorial Policies and the Editorial Policy Checklist.

Statistics

For all statistical analyses, confirm that the following items are present in the figure legend, table legend, main text, or Methods section.

n/a	Confirmed	
☐	☒	The exact sample size (*n*) for each experimental group/condition, given as a discrete number and unit of measurement
☐	☒	A statement on whether measurements were taken from distinct samples or whether the same sample was measured repeatedly
☐	☒	The statistical test(s) used AND whether they are one- or two-sided<br>*Only common tests should be described solely by name; describe more complex techniques in the Methods section.*
☒	☐	A description of all covariates tested
☒	☐	A description of any assumptions or corrections, such as tests of normality and adjustment for multiple comparisons
☐	☒	A full description of the statistical parameters including central tendency (e.g. means) or other basic estimates (e.g. regression coefficient) AND variation (e.g. standard deviation) or associated estimates of uncertainty (e.g. confidence intervals)
☐	☒	For null hypothesis testing, the test statistic (e.g. *F*, *t*, *r*) with confidence intervals, effect sizes, degrees of freedom and *P* value noted<br>*Give P values as exact values whenever suitable.*
☒	☐	For Bayesian analysis, information on the choice of priors and Markov chain Monte Carlo settings
☒	☐	For hierarchical and complex designs, identification of the appropriate level for tests and full reporting of outcomes
☒	☐	Estimates of effect sizes (e.g. Cohen's *d*, Pearson's *r*), indicating how they were calculated

Our web collection on statistics for biologists contains articles on many of the points above.

Software and code

Policy information about availability of computer code

Data collection	LC-MS: Data from qTOF mass spectrometer (Waters) was collected using MassLynx (v4.2).<br>GC-MS: Data from Agilent 7890B GC system was collected using MassHunter Qualitative Analysis (B.07.00).
Data analysis	Heatmap analysis: multiexperiment Viewer (MeV, v4.9.0) and Java 8.<br>NMR data: MestReNova software (v14.0.0).<br>MALDI-MSI: Bruker SCiLS Lab 2020a and MetaboScape software (Bruker).<br>Local blast: BioEdit software (v7.0.9.0).<br>Phylogenetic analysis: MEGA (v6.0), online conversion ALTER (http://www.sing-group.org/ALTER/) and KaKs_Calculator 2.0.<br>LC-MS analysis: MassLynx (v4.2).<br>GC-MS analysis: MassHunter Qualitative Analysis (B.07.00).<br>Graphs: GraphPad Prism (v7.0).

For manuscripts utilizing custom algorithms or software that are central to the research but not yet described in published literature, software must be made available to editors and reviewers. We strongly encourage code deposition in a community repository (e.g. GitHub). See the Nature Portfolio guidelines for submitting code & software for further information.

Data

Policy information about availability of data

All manuscripts must include a data availability statement. This statement should provide the following information, where applicable:

- Accession codes, unique identifiers, or web links for publicly available datasets
- A description of any restrictions on data availability
- For clinical datasets or third party data, please ensure that the statement adheres to our policy

The data supporting the findings of this work are available within the paper and Supplementary files. A reporting summary for this article is available as a Supplementary Information file. The genome and transcriptome sequence data are available at NCBI BioProject PRJNA542587. Full sequences of CYP82D274 (accession XP_038717985 [https://www.ncbi.nlm.nih.gov/protein/XP_038717985.1/]) and CYP82D263 (accession XP_038716720 [https://www.ncbi.nlm.nih.gov/protein/XP_038716720.1/]) are deposited in GenBank. The databases of TargetP v2.0 (https://services.healthtech.dtu.dk/service.php?TargetP-2.0) and TMHMM v2.0 (https://services.healthtech.dtu.dk/service.php?TMHMM-2.0) are used for data analyses in this study. Source data are provided with this paper.

Human research participants

Policy information about studies involving human research participants and Sex and Gender in Research.

Reporting on sex and gender	No human research participants in this study.
Population characteristics	No human research participants in this study.
Recruitment	No human research participants in this study.
Ethics oversight	No human research participants in this study.

Note that full information on the approval of the study protocol must also be provided in the manuscript.

Field-specific reporting

Please select the one below that is the best fit for your research. If you are not sure, read the appropriate sections before making your selection.

☒ Life sciences ☐ Behavioural & social sciences ☐ Ecological, evolutionary & environmental sciences

For a reference copy of the document with all sections, see nature.com/documents/nr-reporting-summary-flat.pdf

Life sciences study design

All studies must disclose on these points even when the disclosure is negative.

Sample size	For yeast engineering, all presented data represent measurements from 3 biological replicates (independently grown microbial cultures). Since engineering was performed at the cellular level and a large population of individual cells, 3 biological replicates were sufficient to observe metabolite differences and clarify results accuracy. For the metabolite feeding suspension cells and gene overexpression & RNAi, 3 biological replicates in each treatment. For kinetic analysis, 3 biological replicates per substrate concentration group. Sample sizes for experiments were determined based on similar published studies and to provide sufficient statistical power for data analysis (PMID 36050299; PMID 36008399; PMID 32080175).
Data exclusions	No data was excluded in this study.
Replication	For yeast heterologous expression, at least 3 independent transformants were generated for every yeast strain. The independent yeast transformants were cultivated independently at different time periods, to verify the accuracy of results.<br>Relative quantification of metabolites was based on 3 biological replicates represented by individual transformants/lines of yeast or suspension cells.
Randomization	Since Tripterygium wilfordii suspension cells form cell clusters with a diameter of 0.2-0.5 cm, we randomly selected cells from the same parent generation and assigned them to different groups according to experimental requirements.
Blinding	Blinding was not relevant to this study. All samples were extracted and assayed in the same way, without any differences.

Reporting for specific materials, systems and methods

We require information from authors about some types of materials, experimental systems and methods used in many studies. Here, indicate whether each material, system or method listed is relevant to your study. If you are not sure if a list item applies to your research, read the appropriate section before selecting a response.

Materials & experimental systems

n/a	Involved in the study
☒	☐ Antibodies
☒	☐ Eukaryotic cell lines
☒	☐ Palaeontology and archaeology
☒	☐ Animals and other organisms
☒	☐ Clinical data
☒	☐ Dual use research of concern

Methods

n/a	Involved in the study
☒	☐ ChIP-seq
☒	☐ Flow cytometry
☒	☐ MRI-based neuroimaging
